# Haemophilia A in a Female German Shepherd With Homozygosity for the FVIII p.C567Y Variant in Exon 11 of the *F8* Gene

**DOI:** 10.1002/age.70154

**Published:** 2026-06-24

**Authors:** Bertram Brenig, Sabrina Pach

**Affiliations:** ^1^ Institute of Veterinary Medicine, University of Göttingen Göttingen Germany

**Keywords:** F8 gene, German shepherd, haemophilia A, missense variant

## Abstract

Haemophilia A is an extremely rare disorder in females, as the causative *F8* gene is located on the X chromosome. Female carriers, also known as ‘conductors,’ are typically heterozygous and therefore do not show clinical signs of the disease. However, in mild forms of haemophilia A, affected males may survive and mate. In such cases, mating a haemophilic male with a carrier female can result in female offspring who are homozygous for a pathogenic variant and consequently develop haemophilia A. In the present case, a female dog with a mild form of haemophilia A is described. Although it had a markedly increased bleeding tendency, bleeding episodes have so far not been fatal. There were no prior indications of haemophilia A inheritance in either parent. To identify a causative variant in the *F8* gene, whole‐genome sequencing was performed on the affected dog. A homozygous missense variant was identified in exon 11 of the *F8* gene, resulting in an amino acid substitution within the A2 domain of factor VIII—a region critical for the stability and function of the coagulation factor. The same variant has previously been described in an unrelated German Shepherd. Notably, many examined variants along the entire X chromosome of the dog were homozygous. The rediscovery of this variant, more than 10 years after its initial description, underscores the importance of comprehensive genetic diagnostics, especially in rare diseases with atypical inheritance patterns.

Very early on, the German Shepherd bitch (born 13 November 2023) developed significant swellings after a microchip was implanted at 3 months of age. In the following months, even minor injuries repeatedly resulted in extensive haematoma formation and prolonged bleeding. The dog was therefore examined for various coagulation parameters on suspicion of haemophilia. Clinical examination of the coagulation parameters (prothrombin time [PT]; activated partial thromboplastin time [aPTT]) showed a significantly increased aPTT of 21 s (reference range 0–13.5 s), while PT was within the reference range at 8.5 s (reference range 0–10.1 s). The test showed very low residual FVIII activity of 6% (reference range 70%–135%). In contrast, the values for FIX (89%) and von Willebrand factor (102%) were within the reference ranges of 75%–140% and 55%–150%, respectively (Geffre et al. 2010). The laboratory diagnostic values therefore indicated haemophilia A. However, as X‐linked haemophilia A is unlikely in females, molecular genetic analysis of the *F8* gene was performed. A hair root sample from the German Shepherd was provided by the owner. All other DNA samples were sourced from the institute's depository and stored as residual blood samples. The use of residual blood samples was approved by the Ethics Committee under no. E5‐21 (2021‐09‐27). The study complies with German legal and ethical requirements for appropriate animal procedures.

Whole‐genome sequencing was performed on a Illumina NovaSeq6000 according to the manufacturers protocols. The quality of the Illumina fastq files was first checked using FastQC v0.12.1 software. The data was then aligned against the canine reference genome canFam6 using BWA‐MEM (Lindblad‐Toh et al. 2005; Li and Durbin 2009). The resulting BAM‐file was further processed using samtools v1.22.1 and gatkHaploytpeCaller (Danecek et al. 2021; Wilton and Szalay 2023). The vcf file was imported into SVS 8.10.0 and GenomeBrowse (Golden Helix Inc., Bozeman, MT).

Read depth was calculated using samtools with command<depth ‐a>. To determine the degree of ploidy, the average read depth was calculated for each chromosome and each position. The average read depth ranged from 33.2 (CFA9) to 45.18 (CFA6). The X chromosome had an average read depth of 36.09, which was within the range of all diploid autosomes. Monosomy (X0) could therefore be excluded as the cause of homozygosity. Alignment of the *F8* sequences of the dog to canFam6 showed a total of 309 variants (Table S1) including six homozygous variants in the coding region (Table 1).

**TABLE 1:** Variants detected in the *F8* coding region in the haemophilic female German Shepherd.

Position^a^	Exon	cDNA^b^	Amino acid^c^	rs‐number	Effect
g.108036424G>A	1	c.141C>T	p.Asp47=	rs852021679	Synonymous
g.107969072A>G	11	c.1614T>C	p.Asp538=	rs3356053415	Synonymous
g.107968986C>T	11	c.1700G>A	p.Cys567Tyr		Missense
g.107950583C>T	14	c.2943G>A	p.Glu981=	rs851733901	Synonymous
g.107949918G>A	14	c.3608C>T	p.Pro1203Leu	rs852844707	Missense
g.107931995G>A	15	c.5292C>T	p.Asn1764=	rs852651766	Synonymous

^a^ Genomic positions correspond to NC_006621.4 (canFam6).

^b^ cDNA positions correspond to NM_001003212.1.

^c^ Amino acid positions correspond to ENSCAFP00000029035.3.

There were two missense variants in exon 11 (g.107968986C>T) and exon 14 (g.107949918G>A). Since the variant in exon 14 (rs852844707) was predicted to be deleterious by SIFT (Sim et al. 2012), we initially suspected this variant to be the cause, although it already had an rs‐number. We therefore tested the presence of this variant in 9 canine control DNAs (4 Dachshund, 1 Weimaraner, 1 Mixed‐breed and 3 Miniature Schnauzer) using PCR with primers cfa_F8_Ex14.5F (5′‐tttttgctaacttggctaatgtc‐3′) and cfa_F8_Ex14.5R (5′‐tccttgtgagagtctgggttg3′) and DNA sequencing (Sanger et al. 1977). However, the variant was detected in the healthy dogs, so it could be ruled out as the cause. To confirm this result, we also examined 722 dog genomes for the variant and detected an allele frequency of 0.462 (Plassais et al. 2019). The allele frequency was also analysed in 1986 canine genomes from the Dog10K consortium (Meadows et al. 2023). In this dataset, an allele frequency of 0.451 was found. Therefore, only the missense variant in exon 11 remained as a possible cause of haemophilia A.

This variant was not detected in the 722 or 1986 dog genomes, nor in the previously mentioned control DNAs, using PCR with primers cfa_F8_Ex11F (5′‐aaaattcgtaaagcccttgaaa‐3′) and cfa_F8_Ex11R (5′‐ggctatagggggatgtactctg‐3′). The read depth at position NC_006621.4:g.107968986C>T in the affected bitch was DP = 23, with a genotype quality of GQ = 69 and an allelic depth of AD = 0.23. To further exclude any other potential variants, we also analysed the data using IGV and GenomeBrowse (Robinson et al. 2023). There was no evidence of any structural variants that might explain the cause of haemophilia A.

As this variant had already been detected in a 5‐year‐old male German Shepherd (OMIA 000437‐9615), it seemed very likely that it was also the cause of haemophilia A in the female dog (Christopherson et al. 2014; Nicholas and Tammen 2023).

According to the CDC Hemophilia A Mutation Project (CHAMP) Mutation List of *F8* variants, there are three variants in humans at amino acid position 567 (two missense and one deletion) (Payne et al. 2013). The two missense variants lead to a mild form of haemophilia A, while the deletion, which causes a frameshift, results in severe haemophilia A. Position ENSCAFP00000029035.3:p.Cys567Tyr is located in the A2 domain, which is part of the heavy chain of FVIII and is essential for its activity (Pittman et al. 1992). Given the position of the variant described here within the A2 domain and its significance for FVIII activity, the previously demonstrated correlation between variants at this position in humans, the absence of this variant in the dog genomes analysed, and the report of a haemophilic male German Shepherd harbouring the same variant, it can be concluded that this variant is the cause of haemophilia A in the present case. In addition, the Animal Variant Classification Guidelines (AVCG) were used for the interpretation and classification of the NC_006621.4:g.107968986C>T variant (Boeykens et al. 2024). As shown in Table S2 the variant was classified as pathogenic.

## Author Contributions

**Sabrina Pach:** investigation, writing – review and editing, validation, visualization. **Bertram Brenig:** conceptualization, investigation, funding acquisition, writing – original draft, validation, visualization, writing – review and editing, project administration, data curation, supervision, resources.

## Conflicts of Interest

The authors declare no conflicts of interest.

## Supporting information


**Table S1:** Variants in the F8 gene region in the German Shepherd bitch.

**Table S2:** Classification of the NC_006621.4:g.107968986C>T variant according to the AVCG criteria (Boeykens et al. 2024).

## Data Availability

WGS data from the affected German Shepherd have been submitted to NCBI with accession number PRJNA1400790.
